# Endoscopic submucosal dissection for early cancer using the water pressure method in the remnant rectum after Hartmann’s procedure

**DOI:** 10.1055/a-2690-1982

**Published:** 2025-09-18

**Authors:** Kumi Itami, Takaaki Yoshikawa, Takeshi Mori, Kazuto Kajimoto, Shujiro Yazumi

**Affiliations:** 1 566610 Department of Gastroenterology and Hepatology, Kitano Hospital Medical Research Institute, Osaka, Japan

Endoscopic submucosal dissection (ESD) for colorectal neoplasms, including laterally spreading tumors, has been widely performed 1 . However, ESD has rarely been reported for neoplasms located in the remnant rectum after Hartmann’s procedure. Herein, we report a successful ESD for early rectal cancer using the water pressure method.

A 74-year-old man was referred to our department due to high uptake of 18F-fluorodeoxyglucose in the rectum. He had undergone Hartmann’s procedure for rectosigmoid colon cancer nine years earlier. Colonoscopy revealed a 40-mm, 0–Is + IIa villous lesion located in the Rb, 2 cm from the anal verge ( Fig. 1 ). Narrow-band imaging partially revealed atypical vascular structures consistent with JNET classification type 2B, suggesting the presence of carcinoma in villous adenoma ( Fig. 2 ).

**Fig. 1:**
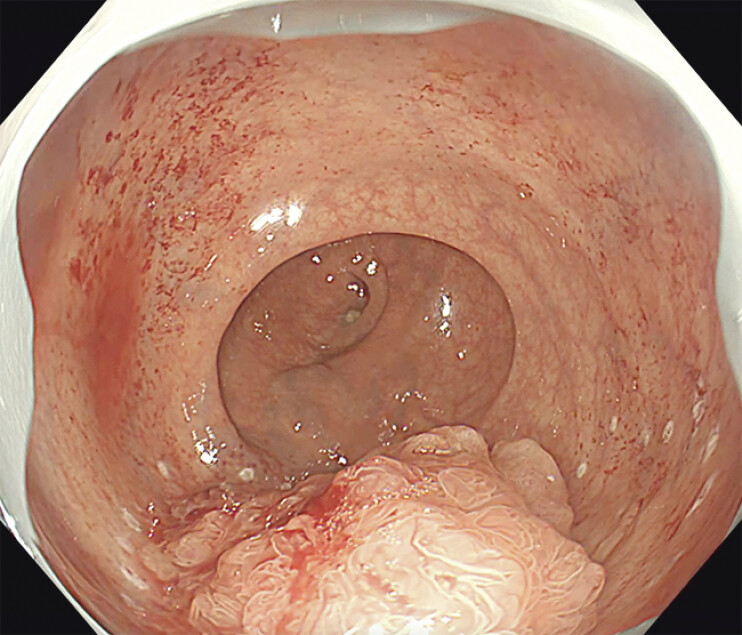
Colonoscopy revealed a 40-mm, 0–Is + IIa villous lesion located in the Rb, 2 cm from the anal verge.

**Fig. 2:**
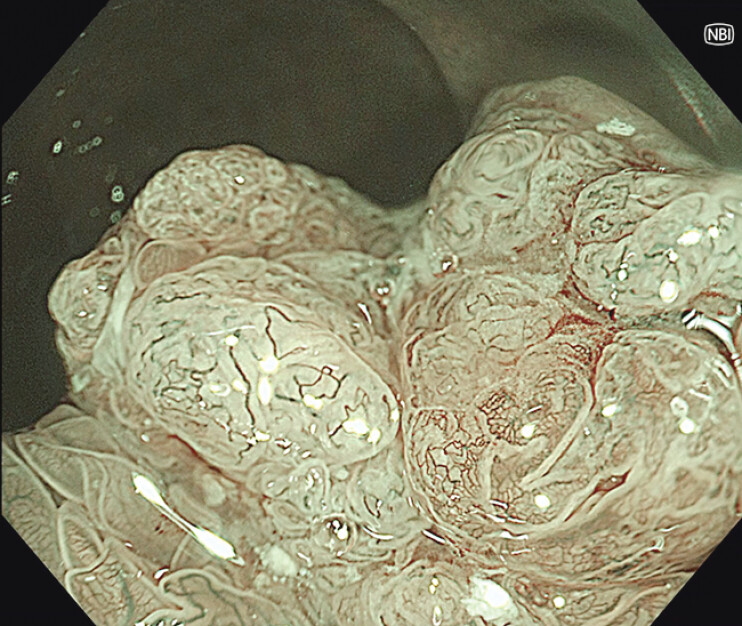
Narrow-band imaging partially revealed an atypical vessel structure consistent with JNET 2B.

When performing ESD ( Video 1 ), even slight CO _2_ insufflation caused abdominal pain and oozing from the fragile mucosa. After mucosal incision and trimming, submucosal dissection was performed using the water pressure method with a short ST hood (DH-28GR; Fujifilm, Tokyo, Japan) without CO _2_ insufflation ( Fig. 3 ). The water pressure method relieved abdominal pain and allowed safe dissection by inflating the atrophic submucosal layer with water. CO₂ insufflation was used in combination with the water pressure method when the dissection reached a stage where traction by gravity was utilized. En bloc resection was accomplished without any complications ( Fig. 4 ). Pathological examination confirmed carcinoma in a villous adenoma with negative margins.

The water pressure method was utilized for endoscopic submucosal dissection in the remnant rectum, relieving abdominal pain and mucosal damage.Video 1

**Fig. 3:**
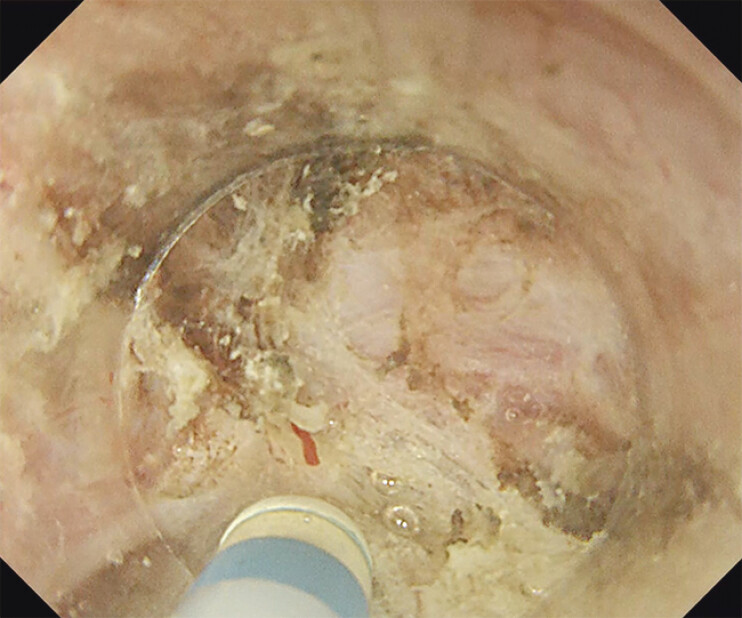
Submucosal dissection was performed using the water pressure method.

**Fig. 4:**
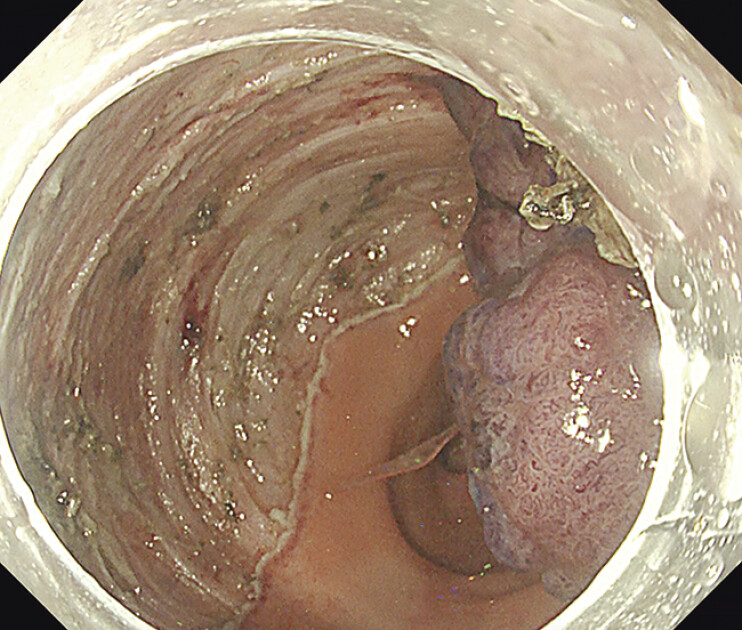
Curative resection was accomplished.

A standardized technique of ESD in the remnant rectum has not yet been established. Insufflation of gas in such a closed cavity readily increases the internal pressure, inducing abdominal pain and mucosal damage. The water pressure method, which is often used in duodenal and colorectal ESD 2 , ensures visibility at low pressure 3 . This method can be a useful technique for safe ESD in closed, narrow spaces, such as the remnant rectum.

Endoscopy_UCTN_Code_TTT_1AQ_2AD_3AZ

## Notes

Endoscopy E-Videos https://eref.thieme.de/e-videos
*E-Videos* is an open access online section of the journal *Endoscopy* , reporting on interesting cases and new techniques in gastroenterological endoscopy. All papers include a high-quality video and are published with a Creative Commons CC-BY license. Endoscopy E-Videos qualify for HINARI discounts and waivers and eligibility is automatically checked during the submission process. We grant 100% waivers to articles whose corresponding authors are based in Group A countries and 50% waivers to those who are based in Group B countries as classified by Research4Life (see: https://www.research4life.org/access/eligibility/ ). This section has its own submission website at https://mc.manuscriptcentral.com/e-videos .
